# High Body Mass Index Is Associated with the Extent of Muscle Damage after Eccentric Exercise

**DOI:** 10.3390/ijerph15071378

**Published:** 2018-07-01

**Authors:** Jooyoung Kim, Wi-Young So

**Affiliations:** 1 Sports, Health, and Rehabilitation Major, College of Physical Education, Kookmin University, Seoul 02707, Korea; hirase1125@hanmail.net; 2 Sports and Health Care Major, College of Humanities and Arts, Korea National University of Transportation, Chungju-si 27469, Korea

**Keywords:** body mass index, creatine kinase, eccentric exercise, muscle damage

## Abstract

*Purpose*: The present study aimed to investigate the effects of body mass index (BMI), which is an obesity index, on the change in the muscle damage index after eccentric exercise. *Methods*: Forty healthy male university students participated in this study and were classified into normal (BMI 18.5–22.9 kg/m^2^, *n* = 20) and high BMI groups (BMI ≥ 25 kg/m^2^, *n* = 20). For eccentric exercise, a modified preacher curl machine was used. Participants performed two exercise sets with 25 repetitions in each set. With regard to the muscle damage index, maximum strength, muscle soreness, and the creatine kinase (CK) level were measured. *Results*: Loss of maximum strength, muscle soreness, and the CK level were higher in the high BMI group than in the normal BMI group (*p* < 0.05, *p* < 0.05, and *p* < 0.01, respectively). *Conclusions*: In conclusion, BMI is one of the potential factors related to muscle damage after eccentric exercise.

## 1. Introduction

Obesity involves the excessive accumulation of fat in the body, and it is one of the problems threatening health [1]. Exercise is well known to reduce many of the causes of obesity and its complications [2]. However, it is also well known that muscle damage can be caused by repetitive high-intensity exercise, especially eccentric exercise that is unfamiliar [3]. Muscle damage generally involves a decrease in maximum strength, delayed onset muscle soreness (DOMS), and increases in the blood levels of muscle proteins, such as creatine kinase (CK) and myoglobin [4].

Previous studies reported that the muscle damage index after eccentric exercise can differ according to the degree of obesity [5,6]. Paschalis et al. reported a remarkable decrease in maximum strength and remarkable increases in DOMS and blood CK levels after eccentric exercise in obese individuals with more than 31% average body fat when compared to the findings in normal individuals [5]. Additionally, Knoblauch et al. reported that cell membrane damage in muscles was higher in obese rats than in normal rats [6]. These findings indicate that a higher degree of obesity is associated with more muscle damage after eccentric exercise. Although there are many studies about the intervention effects of exercise in obese individuals, studies about the relationship between exercise-induced muscle damage and obesity are limited. Thus, the present study aimed to investigate the effects of BMI on the change in the muscle damage index after eccentric exercise.

## 2. Methods

### 2.1. Subjects

This study included 40 healthy male university students. The inclusion criteria were as follows: no regular resistance exercise in the last 6 months, no musculoskeletal disease, and no use of any drug or dietary supplement. The participants were provided with enough information about the study, and they voluntarily signed an informed consent form that was approved by our institutional review board. The participants were divided into the following 2 groups according to the BMI criteria of the WHO Western Pacific Regional Office: normal BMI group (BMI 18.5–22.9 kg/m^2^, *n* = 20) and high BMI group (BMI ≥ 25 kg/m^2^, *n* = 20) [7]. The physical characteristics of the participants are presented in Table 1. The study was conducted in accordance with the Declaration of Helsinki, and the protocol was approved by the Ethics Committee of Kookmin University.

### 2.2. Eccentric Exercise

The eccentric exercise in this study was performed with a modified preacher curl machine (Kookmin University, Seoul, Korea). Each participant began the exercise with the arm fully flexed (elbow joint angle, 90°). The investigator pulled down on a lever, forcing the participant’s arm into an extended position (angle, 0°) as the participant resisted. Muscle contraction of the non-dominant arm was performed for 3 s, and the rest period between muscle contractions was 12 s. Participants performed 2 exercise sets with 25 repetitions in each set, and the rest period between sets was 5 min. For the eccentric exercise protocol, we referred to a previous study by Clarkson et al. [8]. The participants were instructed to intake enough water before and after eccentric exercise, and urine color was continuously checked after eccentric exercise, as dark urine is considered an indicator of rhabdomyolysis. However, no participant showed dark urine.

### 2.3. Maximum Strength

Maximum strength was measured using a strain gauge (Jackson Strength Evaluation System Model 32628CTL; Lafayette Instrument Co., Lafayette, IN, USA) attached to the modified preacher curl machine (Kookmin University, Seoul, Korea). When the participants pulled the pad of the preacher curl machine toward themselves as strongly as possible after placing the elbow joint at 90°, strength was measured and recorded [8,9,10]. A total of 3 maximum strength values were considered, and the mean value was used.

### 2.4. Muscle Soreness

Muscle soreness was measured using a visual analogue scale (VAS). The scale included a 100-mm line, with the left end (0 mm) representing no soreness and the right end (100 mm) representing the most severe soreness. The reliability of the VAS has been demonstrated in advanced studies [11,12]. The researcher asked the participants to indicate the degree of current pain, and the participants made a vertical marking on the VAS according to the perceived pain after repeating elbow flexion and extension in the standing position [13].

### 2.5. Creatine Kinase

About 5 mL of blood was obtained from the brachial vein to assess the CK level. Blood was collected from 8:00 to 8:30 a.m. (fasting status) after arriving at the laboratory. After 15 min of rest, blood was collected in the supine position. Additionally, blood was collected before exercise, and 24, 48, 72, and 96 h after exercise. The participants were told not to perform vigorous or high intensity physical activity in order to minimize the factors that could affect the blood sample. The participants filled a physical activity diary to monitor their daily physical activity.

The obtained blood samples were stored in Vacutainer SST (BD Vacutainer SST™ Tubes, Fisher Scientific, Loughborough, UK), and serum was separated by centrifugation at 2500–3000 rpm for 10 min. The separated serum was stored in a microtube placed in an ultra-low temperature (−80 °C) freezer (DFU-374CE, Operon Co., Ltd., Gimpo, Korea). CK was analyzed with a specific kit (AceChem CK Kit, YD-Diagnostics Corp., Yongin, Korea) using an automatic biochemistry analysis system (Miura One, I.S.E. S.r.l., Guidonia, Italy). On assessing the reliability of CK measurement, the mean correlation coefficient (R-value) was 0.91.

### 2.6. Statistical Analysis

Based on repeated analysis of variance measures (2 × 6 design), an anticipated statistical power of 0.80, and an alpha error probability of 0.05 with an effect size of 0.80, a sample size of 27 participants was determined to be necessary (G-power program 3.1.3, Kiel, Germany) [14,15]. Considering the possibility of withdrawal from the study, 40 participants were included.

Data are expressed as mean and standard deviation. Repeated-measures analysis of variance was used to analyze the interaction effects between the groups and time. An independent samples *t*-test was used to analyze the difference in groups according to the time when an interaction was identified. All analyses were performed using statistical analysis software (SPSS ver. 21.0, IBM Corp., Armonk, NY, USA). Statistical significance was set at a *p*-value < 0.05.

## 3. Results

Both the normal and high BMI groups showed significant time effects with regard to the maximum strength (F = 116.014, *p <* 0.001), muscle soreness (F = 74.467, *p <* 0.001), and CK level (F = 9.104, *p <* 0.01), indicating that the eccentric exercise protocol was well adapted in this study. After eccentric exercise, the maximum strength (F = 3.057, *p =* 0.026), muscle soreness (F = 2.487, *p =* 0.046), and CK level (F = 6.738, *p =* 0.009) showed significant interaction effects between the groups and time according to BMI. The high BMI group showed a greater decrease in maximum strength immediately after exercise when compared to the finding in the normal BMI group, and recovery at 24 (*t* = −2.122, *p <* 0.05), 48 (*t* = −2.219, *p <* 0.05), 72 (*t* = −2.882, *p <* 0.01), and 96 h (*t* = −2.273, *p <* 0.05) after exercise was slower in the high BMI group (Table 2). Muscle soreness increased after exercise in both groups; however, the high BMI group showed higher muscle soreness at 72 h (*t* = 2.036, *p <* 0.05) after exercise when compared to the finding in the normal BMI group (Table 3). Moreover, the CK level also increased after exercise in both groups; however, the high BMI group showed a higher CK level at 24 (*t* = 2.353, *p <* 0.05), 48 (*t* = 3.835, *p <* 0.001), 72 (*t* = 4.053, *p <* 0.001), and 96 h (*t* = 2.768, *p <* 0.05) after exercise when compared to the findings in the normal BMI group (Table 4).

## 4. Discussion

This study attempted to investigate the effects of BMI on the muscle damage index after eccentric exercise. The BMI is one of the indexes for evaluating body composition [16]. According to the BMI classification of the WHO Western Pacific Regional Office [7], a high BMI of ≥25 kg/m^2^ can be considered as obesity. Some studies have reported that obesity might increase muscle damage after exercise [5,17]. The present study also showed similar findings. We found that high BMI was associated with higher loss of maximum strength, muscle soreness, and CK after eccentric exercise when compared with normal BMI.

The change in maximum strength after eccentric exercise represents the magnitude of muscle damage and recovery of muscular function [4,13]. In this study, maximum strength showed significant differences between the high BMI and normal BMI groups from immediately after exercise to 96 h after exercise, indicating that high BMI was associated with high muscle damage and slow recovery. In a previous human study that involved biopsy for the analysis of tissue, it was reported that obese individuals had a high proportion of type II muscle fiber [18,19], which is associated with increased muscle damage during eccentric exercise [5]. Obesity can decrease muscular regeneration and muscular function recovery after damage [20]. Some studies have suggested that obesity decreases satellite cell activation, anabolic signaling, insulin sensitivity, and 5′-AMP-activated protein kinase (AMPK) activity, which are required for muscular regeneration [20,21,22,23]. In addition, inflammatory responses in obese individuals are related to a decrease in muscular regeneration [24]. It is known that lipocyte accumulation in obese individuals causes chronic low-grade inflammation through increases in the levels of pro-inflammatory cytokines, such as interleukin-6 and tumor necrosis factor-alpha [25]. These inflammatory responses can interfere with the processes involved in muscle protein synthesis [21].

Inflammatory responses are thought to have influenced the change in muscle soreness in this study. Inflammatory responses are known to be associated with muscle soreness [26]. Pre-existing chronic low-grade inflammation in patients with high BMI might have increased muscle soreness through an increase in acute inflammation after eccentric exercise. In the study by Paschalis et al. [5], muscle soreness in the recovery phase after eccentric exercise was higher in obese individuals than in normal individuals. However, the present study and the study by Paschalis et al. [5] did not measure inflammation response-related factors. Thus, this study could not determine the inflammation state of the patients with high BMI and could not assess whether the inflammation state directly affected the increase in muscle soreness after eccentric exercise. Future studies will be needed to obtain further information.

CK is an indirect marker of cell membrane damage in muscles after exercise [27]. It has been reported that cell membrane damage after exercise is higher in obese individuals than in normal individuals; thus, the CK level is higher in obese individuals [17]. This finding is associated with the characteristics of obese individuals. It is known that obese individuals have a higher CK level in the resting condition [17], which is related to the presence of high body fat [28]. In the present study, the high BMI group showed higher CK levels at 24, 48, 72, and 96 h after exercise when compared to the findings in the normal BMI group.

Some studies have reported that conditions in obese individuals, such as dyslipidemia, can change the composition of the cell membrane [6,29]. Indeed, it has been demonstrated that saturation of the fatty acyl chain in the muscle cell membrane increased with high fat [30]. Additionally, cell membranes containing saturated acyl chains have been shown to have decreased mobility owing to increased packing density and rigidity [31,32]. These changes are believed to possibly increase the resting CK level and make the cell membrane vulnerable to damage during eccentric exercise. However, these assumptions could not be clarified as this study did not examine the blood lipid profile and condition of dyslipidemia in the participants.

The present study has several limitations. This study only assessed maximum strength, muscle soreness, and CK changes after eccentric exercise, which limited the evaluation of the potential mechanisms by which BMI influences muscle damage. Furthermore, markers for muscle regeneration and inflammation, such as interleukin-6 and tumor necrosis factor alpha, and the blood lipid profile should be examined. Moreover, without dual-energy X-ray absorptiometry or the skinfold method, it is impossible to determine the lean mass content of participants from both groups, particularly those from the high BMI group, who may have been included in this group because of a large amount of lean mass, which would dramatically influence associated strength measurements. However, there was no significant difference in maximum strength between the groups at baseline in this study. Further well-designed studies are necessary.

## 5. Conclusions

Participants with high BMI showed high loss of maximum strength as well as high muscle soreness and CK levels after eccentric exercise. The findings of this study indicated that BMI is one of the potential factors related to muscle damage after eccentric exercise.

## Figures and Tables

**Table 1:** Characteristics of the participants.

	Normal BMI (*n* = 20)	High BMI (*n* = 20)	*p* Value
Age (years)	22.7 ± 2.2	23.4 ± 2.6	0.399
Height (cm)	176.2 ± 6.9	176.7 ± 4.8	0.771
Weight (kg)	66.1 ± 5.5	83.9 ± 7.0	<0.001 ***
BMI (kg/m^2^)	21.3 ± 1.0	26.8 ± 1.6	<0.001 ***
Maximal Strength (Ib)	226.5 ± 46.4	207.4 ± 42.2	0.181

Values are means ± standard deviation; BMI, body mass index. *** *p* < 0.001; tested by independent *t*-test.

**Table 2:** Changes in the maximum strength according to the body mass index after eccentric exercise.

Unit: %	Pre	Post	24 h	48 h	72 h	96 h
Normal BMI (*n* = 20)	100.0 ± 0.0	52.1 ± 16.4	62.4 ± 20.2	65.7 ± 21.8	73.0 ± 15.0	80.2 ± 14.9
High BMI (*n* = 20)	100.0 ± 0.0	36.2 ± 12.5 **	49.8 ± 17.0 *	51.6 ± 17.9 *	56.2 ± 21.3 **	65.5 ± 24.6 *

Values are means ± standard deviation. * Significant between group (*p* < 0.05); ** Significant between group (*p* < 0.01); BMI, body mass index.

**Table 3:** Changes in muscle soreness according to the body mass index after eccentric exercise.

Unit: mm	Pre	24 h	48 h	72 h	96 h
Normal BMI (*n* = 20)	0.0 ± 0.0	36.6 ± 19.2	37.5 ± 21.4	27.7 ± 22.7	18.5 ± 20.3
High BMI (*n* = 20)	0.0 ± 0.0	39.7 ± 16.3	50.2 ± 20.6	41.6 ± 20.5 *	30.4 ± 20.9

Values are means ± standard deviation. * Significant between group (*p* < 0.05); BMI, body mass index.

**Table 4:** Changes in the creatine kinase level according to the body mass index after eccentric exercise.

Unit: U/L	Pre	24 h	48 h	72 h	96 h
Normal BMI (*n* = 20)	115.4 ± 42.3	164.1 ± 81.3	238.4 ± 151.4	545.1 ± 789.6	1020.3 ± 1712.1
High BMI (*n* = 20)	225.5 ± 288.3	561.7 ± 751.1 *	3040.5 ± 3246.2 ***	6181.2 ± 6170.4 ***	12,281.0 ± 18,114.2 *

Values are means ± standard deviation. No significant between group at pre-exercise; * Significant between group (*p* < 0.05); *** Significant between group (*p* < 0.001); BMI, body mass index.
